# Barriers and Enablers of Delivering Asthma Self‐Management to Those With Intellectual Disabilities: A Scoping Review

**DOI:** 10.1111/jar.70155

**Published:** 2025-11-25

**Authors:** Louise Allan, Nicola Roberts, Nicola Ring, Lisa O'Leary

**Affiliations:** ^1^ School of Health and Social Care, Edinburgh Napier University Edinburgh Scotland UK

**Keywords:** action plan, asthma, carers, intellectual disability, respiratory health, self‐management

## Abstract

**Background:**

Respiratory‐related illness including asthma is a leading cause of avoidable higher mortality in those with intellectual disabilities. International guidelines stress the importance of good self‐management in avoiding asthma‐related deaths but give no guidance on how this is achieved with this vulnerable population.

**Method:**

A scoping review of published research to identify barriers and facilitators to promoting asthma self‐management in people with intellectual disabilities.

**Results:**

Only six studies published from 2015 to 2022 met study inclusion criteria. Studies commonly reported that the education of patients and caregivers was critical, with a lack of education being a barrier to good asthma control. Three studies also highlighted the importance of caregivers and support workers in helping those with intellectual disabilities to self‐manage their asthma.

**Conclusions:**

This review highlights the paucity of research in this area. Further research is urgently needed to improve asthma self‐management in those with intellectual disabilities thereby reducing asthma‐related deaths.

## Lay Summary

- This review highlights the lack of research in asthma self‐management in those with intellectual disabilities.
- Further research is important to improve asthma self‐management in this group thereby reducing asthma‐related deaths and improving quality of life.
- Education of patients and caregivers is important and this group are important in helping those with intellectual disabilities self‐manage their asthma.

## 1 Introduction

People with intellectual disabilities have been shown to have poor health outcomes and are more likely to die from causes that could be avoided with better health care, compared to the general population (O'Leary et al. 2018). This group has higher incidences of morbidity and avoidable mortality and young people with intellectual disabilities are nine times more likely to die from treatable causes than the general population (Rydzewska et al. 2025).

Recent evidence has indicated that individuals with intellectual disabilities are more likely to have poorer respiratory health than those without intellectual disabilities, with significantly higher rates of respiratory‐associated death than in the general population (Truesdale et al. 2021). Evidence also demonstrates that respiratory‐related illness is a leading cause of mortality in people with intellectual disabilities (O'Leary et al. 2018; Truesdale et al. 2021; Rydzewska et al. 2025).

Asthma is a prevalent respiratory condition; it is a major chronic inflammatory disease that affects children and adults (WHO 2022). Asthmatics experience swelling of the airways in the lungs causing symptoms such as cough, wheeze, shortness of breath and chest tightness, all of which can affect people's quality of life (WHO 2022). Asthma can be managed effectively with bronchodilators and steroid inhalers and by avoiding certain things that can act as triggers and exacerbate symptoms (WHO 2022). Research from Smith et al. (2025) demonstrated that young people with an intellectual disability had higher asthma prevalence compared to the age‐matched general population. For those with asthma having an intellectual disability can complicate health outcomes (Sturdy et al. 2002). Sturdy et al. (2002) found that of adults admitted to the hospital due to asthma those who died were more likely to have an intellectual disability. British Thoracic Society (BTS) clinical guidance lists intellectual disabilities as a risk factor for fatal asthma BTS (2025). Smith et al. (2025) found that young people with asthma have longer emergency admissions for asthma compared to the age‐matched population. A systematic review of international studies by Dunn et al. (2018) reported that in seven included studies there were higher rates of hospital admissions for asthma in those with intellectual disabilities than in the general population across all ages.

It is therefore essential to determine how to improve health outcomes for respiratory diseases, such as asthma, in people with intellectual disabilities to avoid preventable premature death as well as hospital admissions. In the UK both the National Institute for Health and Excellence (NICE), British Thoracic Society and Scottish Intercollegiate Guidelines Network (BTS/SIGN) have joint guidance on how to manage asthma. The guidelines state that adults, young people and children should be offered a self‐management programme with a personalised action plan, along with self‐management education (BTS/SIGN/NICE 2024). Despite being internationally recommended as good practice in asthma care for over 20 years, the promotion and use of asthma action plans is suboptimal across all patient groups (Ring et al. 2015). A systematic review of action plan use found a variety of barriers and facilitators in self‐management of asthma, which cover social support to healthcare access, as well as patient education (Miles et al. 2017). This evidence is for the general population and does not include any reference to those with intellectual disabilities who already face additional barriers in accessing health care. There has been some published research on asthma self‐management in people with intellectual disabilities. Davis et al. (2015) looked at inhaler use and knowledge of asthma medication recommending that healthcare professionals promote action plans and educate patients about asthma medication side effects.

To investigate self‐management of asthma in those with intellectual disabilities and inform further research current evidence was identified. The specific aim of the study was to conduct a scoping review of evidence on enablers and barriers to self‐management of asthma in people with intellectual disabilities, including carers (paid or unpaid), family, and health professionals.

## 2 Methods

An initial preliminary search of MEDLINE, the Cochrane Database of Systematic Reviews and JBI Evidence Synthesis found that no current systematic reviews or scoping reviews on the topic were identified. Consideration was given to which type of review would be suitable for this study. Scoping reviews are appropriate for identifying evidence gaps in a topic area (Arksey and O'Malley 2005) and as little research currently exists in this area a scoping review approach was chosen. A methodological framework developed by Arksey and O'Malley (2005) was utilised for this scoping review.

### 2.1 Stage 1: Identification of the Research Question

The study aim was to conduct a scoping review of evidence on enablers and barriers to self‐management of asthma in people with intellectual disabilities. In the context of this review self‐management also included support from carers (unpaid or paid), family, and other professionals.

### 2.2 Stage 2: Search Strategy (Identifying Relevant Studies)

**FIGURE 1:**
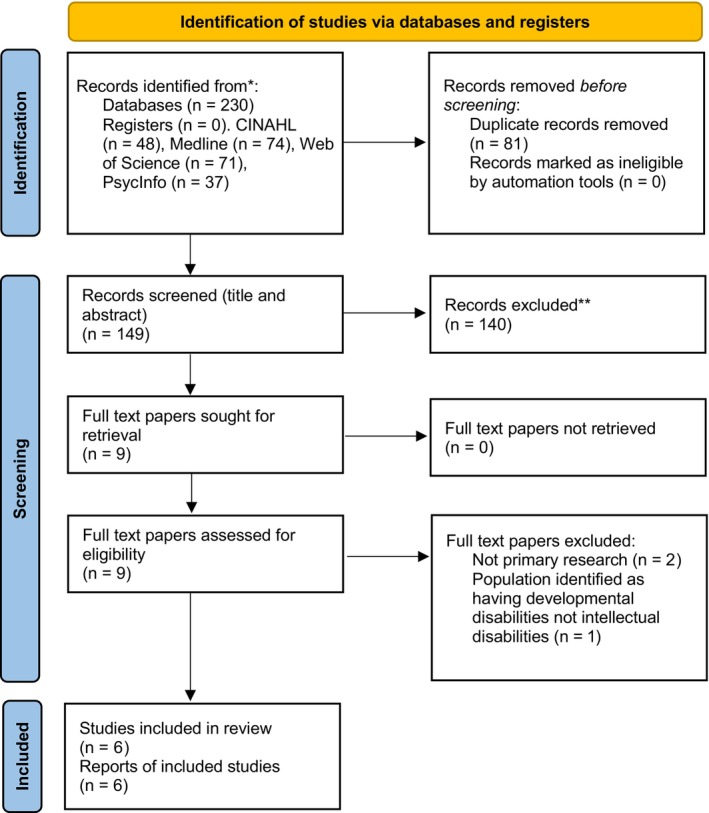
PRISMA flow diagram (Page et al. 2021).

The scoping review was conducted in accordance with the JBI methodology for scoping reviews (Peters et al. 2020), using the Preferred Reporting Items for Systematic reviews and Meta‐Analysis extension for Scoping Reviews (PRISMA‐ScR) Checklist (Tricco et al. 2018). Searches were run (November 2024) as per the initial stages of the PRISMA design (Figure 1). A range of databases was searched: Medline, Web of Science, CINAHL, and PsycInfo. No restrictions on the date of publication were placed. All studies reporting primary research (all types), service evaluations and quality improvements were included. No unpublished literature was included due to time and resource constraints.

The PCC approach (Peters et al. 2020) was used to inform the search strategy and inclusion/exclusion criteria. ‘Population’ was identified as individuals (of all ages) with intellectual disabilities and asthma, and their families/carers. It is important to note that learning disability is often used interchangeably with intellectual disability in research, and this term was included in the search strategy. ‘Concept’ was specified as self‐management of asthma. For the purposes of the review self‐management is taken to mean where people with intellectual disabilities are managing asthma on their own and/or with the help and support of families/carers or other professionals. The ‘Context’ component was any geographical location and any setting.

A three‐tiered search strategy was employed. This strategy was informed by PCC elements identified above. The search strategy, including all identified keywords and index terms, was adapted for each included database.

### 2.3 Stage 3: Study Selection

The following inclusion criteria were applied when screening the titles and abstracts in the databases:

- English Language
- Population: all ages, individual with intellectual disabilities as participants, including carers/families of this population as well as professionals working with this population
- Settings: all settings, all regions
- Study design: Primary research (qualitative and quantitative), service evaluation and quality improvement reports
- Subject: Asthma self‐management

The following exclusion criteria were applied when screening the titles and abstracts:

- Full paper not accessible or not available in English
- Population: studies where the proportion of participants with intellectual disabilities is unclear or < 50% of sample (if not reported separately)
- Study design: Not primary research, service evaluation or quality improvement studies, for example, reviews, editorials and narrative reports were excluded

The selection process comprised four stages in line with PRISMA guidelines (Tricco et al. 2018). Following the search, all identified citations were collated and duplicates removed. After preliminary screening against the study inclusion criteria, these inclusion and exclusion criteria were reviewed with the wider team to determine if criteria needed to be refined. The titles and abstracts of selected citations were then assessed in detail against the inclusion and exclusion criteria by the primary author [L.A.] and an independent reviewer [N.R.]. No disagreements were found between the primary author and independent reviewer. Reasons for exclusion of sources of evidence at full text that did not meet the inclusion criteria were recorded (PRISMA; Figure 1).

### 2.4 Stages 4–5: Data Charting, Collating, Summarising and Reporting

A data extraction template was developed to extract key study characteristics, for example, author(s), publication year, country where the study was published or conducted, aims/purpose of the study, population (including the level of intellectual disability of participants), sample size (if applicable) and methods (Table 1). The outcomes and results of the papers were summarised (Table 1). Data extraction focused on barriers and enablers of asthma self‐management. Extraction was primarily performed by the primary author [L.A.] and cross‐checked for accuracy by a second reviewer [N.R.]. Data extraction was conducted by using thematic analysis and using tables (Tables 1 and 2). After the data was extracted the whole team [L.A., N.R., N.R. and L.O.] met to discuss the results and come to a consensus as per guidance by Mak and Thomas (2022).

**TABLE 1:** Literature table.

1. Author(s), year, country	3. Aims	4. Population, sample size and recruitment	5. Study design and method of data collection	6. Method of data analysis	7. Key results
Cole, E. 2019, UK	Not stated	28 asthma patients with an intellectual disability (no demographic information provided)	Not stated—clinical evaluation paper Asthma clinic run by respiratory nurse to support patients 6 month long	Not stated	Evaluation showed a 25% reduction in this group of patients accessing the emergency department, ambulance services and out of hours GP services Clinic approved by business management team at end of trial and rolled out to all respiratory patients Not clear if due to empowerment, greater understanding of self‐management, compliance or combination ‘Of the people who come to this clinic, 90% would not manage the asthma plan by Asthma UK’
Davis et al. 2016a, Australia	To investigate the level of understanding of inhaled asthma medication use of people with intellectual disabilities, in the context of asthma self‐management	Adults with ID with a diagnosis of asthma Seven lived independently, six lived in group homes, three with family and one in a large residential centre Ages 25–68. Sixty‐five percent of females Level of intellectual disability varied (mild to moderate) Sample size = 17 participants Recruitment: Government and non‐government organisations providing support services invited to identify potential participants	Exploratory observational study using qualitative methods based on thematic framework approach Data collected using one‐on‐one interviews at the location where the person accessed health care, the offices of the support organisation, or the participant's home	Background information was summarised descriptively using SPSS. All other data analysed qualitatively (open coding and axial coding)	All participants in this study self‐administered their asthma medications Level of autonomy for medication acquisition and use was varied The means by which participants in this study acquired information about asthma and its treatment varied, with some participants obtaining information personally from a pharmacist, and others relying on caregivers or other personnel to inform them
Davis et al. 2016b , Australia	To assess inhaler technique of people with intellectual disabilities, and evaluate the effectiveness of teaching with respect to their individual ability to adopt correct technique	Adults with intellectual disability with a diagnosis of asthma Seven lived independently, six lived in group homes, three with family and one in a large residential centre Age: Median 57. Sixty‐five percent of females. Level of intellectual disability not given Sample size = 17 participants Recruitment: Government and non‐government organisations providing support services invited to identify potential participants	Mixed methods: assessment of inhaler technique quantitatively using checklists and qualitative interview as well as training Research conducted at the location where the person accessed health care, the offices of the support organisation, or the participant's home	Background information summarised descriptively. Inhaler technique analysed quantitatively and qualitatively using deductive approach	Unique cognitive barriers exist in those with intellectual disabilities therefore training of inhaler use requires modification to address this Repeated assessment and instruction during visits with healthcare professionals is recommended for people with intellectual disabilities Training should be extended to caregivers and support workers
Davis et al. 2015, Australia	To explore direct support professionals' (DSPs) experiences with asthma medication management in those with intellectual disabilities	DSPs working in residential sites for those with intellectual disabilities where at least one client had asthma. Sample size = 22 Level of intellectual disability of clients not given Recruitment: direct contact with Government department who distributed information to employees. Non‐governmental organisations were also contacted and asked to provide information to employees	Qualitative study combining empirical and grounded theory approaches using semi‐structured interviews	Analysis performed using strategies from grounded theory. Open coding and concepts identified	Range of involvement in asthma management for clients ranged from supervising self‐administration to giving medications to coping in acute situations Most DSPs indicated that the process by which they would know how to deal with any deterioration in asthma status was to consult the asthma healthcare plan DSPs had to decide the degree to which their clients were able to self‐manage their asthma, and when they needed to step in and assist The level of medication training received by participants was identified as variable
Erickson et al. 2018, USA	To assess the level of understanding of asthma self‐management concepts of family caregivers who provide assistance to people with intellectual disabilities and asthma	Caregivers of adults (18 years+) with intellectual disabilities and asthma. Sample size = 19 Level of intellectual disabilitiy not given Recruitment: Using online medical database potential participants were identified and contacted	Pilot qualitative and quantitative study Data collection was remote. Participants mailed survey packs. Included were Asthma Self‐Management Questionnaire, a standardised checklist of inhaler technique and a survey (comprising open ended questions)	Descriptive statistics were reported—standard deviation for continuous variables and frequencies with percentage for categorical variables. Qualitative data were analysed using a thematic content analysis with inductive coding	Most caregivers had acceptable health literacy, but had low scores on the asthma self‐management and inhaler technique test Over a third of the patients with intellectual disabilities did not have desired control of asthma, potentially due to nonadherence with controller medications One of the first studies to provide evidence of the level of asthma control in patients with intellectual disabilities using a validated measure (validated in study looking at general population, Erickson et al. 1998)
Nabors et al. 2022, USA	To conduct a needs assessment to ascertain professionals' and parents' knowledge of and perceptions about education for self‐management of asthma for children with physical and intellectual disabilities	Professionals (nurses, doctors, other health professionals, teachers) working with and parents of children with physical/intellectual disabilities. Sample size = 498 Level of intellectual disability not given Recruitment: recruitment script sent out by several organisations including American Lung Association, Allergy & Asthma Network	Observational study Remote survey with eight questions assessing education and management of asthma for children with physical and intellectual disabilities on a five‐point scale	Descriptive statistics for demographics calculated in SPSS. Quantitative data from surveys analyses using SPSS including mean comparisons, ANOVAs and a MANOVA	Respondents agreed that children with intellectual disabilities had the ability to manage their asthma but would benefit from education to manage their asthma Respondents' perceptions of the ability of children with intellectual and physical disabilities with mild to moderate delays to learn to manage asthma were higher than those for children with intellectual and physical disabilities with severe delays Teachers were less likely than other professionals to believe that they had the education they needed to teach children with intellectual disabilities how to identify asthma triggers, take medications

**TABLE 2:** Self‐management results in included studies.

Paper	Self‐management mentioned?	Definition of self‐management include in paper?	Results of study relating to self‐management enablers	Results of study relating to self‐management barriers
Cole (2019)	Yes	No	Building a rapport and relationship: first contact at the clinic is mainly fact‐finding and getting to know the patient It is important to know what it is that the patient needs to understand and what makes them tick Easy‐read versions of literature were created, including appointment letters, asthma education leaflets and an easy‐read self‐management plan Patient passports including all of patient's contacts and management plan to help with continuity of care	Patient struggle to understand literature available in normal clinics Difficulties occur maintaining plans when patients have several carers
Davis et al. (2016a)	Yes	Yes ‘.. includes self‐monitoring of symptoms, adherence to the treatment regimen, the ability to demonstrate the correct use of inhalers, and the identification of triggers’	Reminders to use medications that participants reported to be useful included a mobile phone alarm Visual cues, such as the colour and location of the inhaler were also helpful Motivation to self‐manage asthma appeared to be influenced by the level of support that was practically available Participants described the security of having caregiver support when needed All participants self‐administered medication but the majority reported that they relied on their caregivers, family, respite staff or HCPs for acquisition, reminding them to take it or with teaching them how to administer	Concerns regarding side effects, stigma in using medications and inconsistent use of spacer devices, reflected in reported intentional and non‐intentional non‐adherence The complexity involved in correctly using inhalers may also have affected adherence for some users in this study, with several participants reporting that they had experienced physical difficulty with using inhaler devices in the past
Davis et al. (2016b)	Yes	No	Facilitators for educator delivery of inhaler technique education included the use of analogies, being patient, and presence of caregivers	Poor comprehension of breathing processes, lack of attentiveness and poor dexterity Interjection of caregivers in attendance—some instructions put forward by caregivers were counterproductive
Davis et al. (2015)	Yes	Yes ‘… requires a patient with asthma to be able to understand the use of asthma medications (e.g., the use of p.r.n. [as needed], or “reliever,” medication vs. regular, or “preventer,” medication) and to use the correct inhaler technique’	Support workers (DSPs) saw their role as supporting their clients to live as independently as possible DSPs aid clients in medication administration Asthma management plans used by DSPs to know how to deal with deterioration and as a source of information on mediations	DSPS are involved in asthma management but not provided with education and tools to manage asthma in those with intellectual disabilities Lack of training on inhaler technique for DSP Asthma action plans not viewed as fit for purpose by DSPs
Erickson et al. (2018)	Yes	No	Caregivers very important in self‐management of asthma in those with ID Two‐thirds of people with intellectual disabilities depended completely on caregiver to assist them in managing their asthma medication	Most frequently cited barriers to controlling asthma were inadequate caregiver and patient education about the illness as well as knowing and avoiding asthma triggers Most cited barriers by caregivers to using asthma medication safely and effectively were knowing and performing correct inhaler technique and education about medications
Nabors et al. (2022)	Yes	No	78.9% of respondents agreed they had the education needed to teach children with intellectual disabilities about asthma triggers 74.2% agreed that they could teach children with intellectual disabilities to take their medications	Significant number of respondents reported disagreeing that children with intellectual disabilities received the education needed to perform self‐care tasks to manage their asthma

## 3 Results

### 3.1 Study Characteristics

The database search produced 230 potential articles, of which 81 were found to be duplicates. A further 140 articles were excluded based on title and abstract screening (PRISMA Figure 1). Full texts of the remaining nine articles were assessed for eligibility of which three were excluded as they did not meet the inclusion criteria. The remaining six articles were eligible for inclusion in the review. All six studies were primary research and used populations of participants with intellectual disabilities (Cole 2019; Davis et al. 2015, 2016a, 2016b; Erickson et al. 2018; Nabors et al. 2022). The two Davis et al. papers from 2016 are separate studies.

### 3.2 Description of Included Studies

Study designs were varied: two were qualitative interview studies (Davis et al. 2015, 2016a), one was mixed methods using a questionnaire (open‐ended) with a checklist of inhaler technique (Erickson et al. 2018), one was mixed methods using an inhaler technique checklist and interview (Davis et al. 2016b), one was a clinical service evaluation paper (Cole 2019) and one used a remote survey with quantitative data (Nabors et al. 2022). The studies were all relatively recently published between 2015 and 2021. Three of the studies were conducted in Australia (Davis et al. 2016a, 2016b), two in the USA (Erickson et al. 2018; Nabors et al. 2022), and one in the UK (Cole 2019).

### 3.3 Participants

All studies included either participants with some level of intellectual disability with asthma (Cole 2019; Davis et al. 2016a, 2016b) or included the support workers (Davis et al. 2015; Nabors et al. 2022)/carers (Erickson et al. 2018)/family (Nabors et al. 2022) of those with some level of intellectual disability with asthma. Nabors et al. (2022) was the only study where the population with intellectual disability was solely children.

Level of intellectual disability was indicated in only one paper (Davis et al. 2016a). Sample size ranged significantly from 17 (Davis et al. 2016a, 2016b) to 498 (Nabors et al. 2022). All studies included adults with the minimum age of any participants being 19 (Erickson et al. 2018).

### 3.4 Self‐Management and Involvement of Caregivers/Support Workers

All six included studies referred to self‐management (Table 2) but only two studies provided a definition of self‐management (Davis et al. 2015, 2016a). Davis et al. (2016a) stated that self‐management ‘.. includes self‐monitoring of symptoms, adherence to the treatment regimen, the ability to demonstrate the correct use of inhalers, and the identification of triggers’ and Davis et al. (2015) stated self‐management ‘… requires a patient with asthma to be able to understand the use of asthma medications (e.g., the use of as needed, or “reliever,” medication vs. regular, or “preventer,” medication) and to use the correct inhaler technique’. Davis et al. (2015) made a direct link and explicitly stated that the involvement of caregivers or support workers is a component of asthma self‐management. Davis et al. (2015) explored the role of support workers and found that their involvement in asthma management in those with intellectual disabilities ranged from supervising self‐administration of medications, to giving medications and responding to acute situations. Davis et al. (2016a) gathered views of participants on their asthma self‐management and it was found that there is a reliance on caregivers, family and respite staff. Erickson et al. (2018) found that two thirds of those with intellectual disabilities in their study depended completely on their caregiver to assist them in managing their asthma medication. This study (Erickson et al. 2018) explored the knowledge of caregivers of asthma self‐management but linked this to the concept of asthma control rather than asthma self‐management.

### 3.5 Enablers of Self‐Management

All six studies referred to enablers to self‐management of asthma. Three studies identified that support from caregivers and support staff is important (Erickson et al. 2018; Davis et al. 2015, 2016a). The enablers for those with intellectual disabilities will be slightly different for those who self‐administer medication than for those who rely on others to administer it for them. However even in those who self‐administer medication, the support from others is still important as indicated by Davis et al. (2016a). Davis et al. (2016a) found that motivation to self‐manage was influenced by the level of support available to the person with intellectual disabilities. All participants in this study self‐administered their own medication but the majority reported that they relied on their caregivers, family, respite staff or health care professionals for acquisition of medication, reminding them to take it or teaching them how to administer it (Davis et al. 2016a). One participant said ‘sometimes I forget; they remind me, they do, the staff’ referring to caregivers supporting them with their medication (Davis et al. 2016a), this is echoed in Davis's earlier study (Davis et al. 2015) where support workers working with those with intellectual disabilities played a major role in supervising and administering clients' medication. One support worker stated ‘Observe them and make sure they take it; it's their health you're dealing with. It's pretty much the most important thing we do in our job I think’ (Davis et al. 2015). These support workers stated that they relied heavily upon asthma management plans to assist with knowing what to do when a client deteriorates and using them as a source of information about medication (Davis et al. 2015). Nabors et al. (2022) found that almost 80% (*n* = 302) of their participants (families and professionals) felt they had the education they required to teach children with intellectual disabilities about triggers and almost 75% (*n* = 285) felt they were able to teach children to take their medications.

Cole (2019) found in their service evaluation that the presence of a dedicated asthma nurse‐led clinic for 28 patients with intellectual disabilities over a 6‐month long period led to a 25% reduction in patients accessing the emergency department, out‐of‐hours GP services and ambulance services; however this could not be attributed solely to increased self‐management. The authors report that while supporting patients with intellectual disabilities it is important to build a relationship with them (Cole 2019). The study also used an easy‐read version of literature including self‐management plans (Cole 2019).

Davis et al. (2016a) reported that in their study exploring how participants manage their asthma participants found visual cues useful in managing their asthma, with one participant discussing having different colours of inhalers—‘I put this one on the kitchen table; the orange one is beside my bed’ (Davis et al. 2016a). Participants also reported having medication reminders such as mobile phone alarms that are useful (Davis et al. 2016a). Davis et al. (2016b) examined how those with intellectual disabilities use their inhalers and found that the delivery of inhaler training by professionals requires the use of analogies and being patient and found that having a caregiver present during training was beneficial. Cognitive barriers exist in those with intellectual disabilities; therefore training in inhaler use needs tailoring to the participant taken into account.

### 3.6 Barriers to Self‐Management

All six studies reported barriers to the self‐management of asthma. Physical difficulties with using inhalers were reported in two studies (Davis et al. 2016a, 2016b). Erickson et al. (2018) reported that over one third of patients did not have the desired control of their asthma which was potentially attributed to non‐adherence with controller medications. Davis et al. (2016a) reported intentional and non‐intentional non‐adherence with medication, as well as inconsistent use of spacer devices. Education was also a potential barrier to self‐management. Nabors et al. (2022) found that 42% of caregivers and health professionals disagreed with the statement that children with mild‐to‐moderate intellectual disabilities received the education needed to provide self‐care or manage their asthma and this figure increased (to 65%) for children with severe intellectual disabilities.

The barriers to those with intellectual disabilities will be slightly different for those who self‐administer medication than for those who rely on others to administer it for them. None of the studies specifically differentiated between these groups of people, However as expected, the knowledge and skills of support workers and carers were identified as a barrier to self‐management of asthma. Lack of education was highlighted as a problem in all three studies that looked at the role of caregivers, support workers, and health professionals (Davis et al. 2015; Erickson et al. 2018; Nabors et al. 2022). Erickson et al. (2018) found that while most caregivers had acceptable health literacy levels, they scored low on asthma self‐management and inhaler technique tests especially relating to using medications and knowing about and performing correct inhaler technique (Erickson et al. 2018). Davis et al. (2016b) reported that inhaler use training should not focus solely on the patient with intellectual disabilities but should also include their caregivers and/or support workers. Erickson et al. (2018) found that inadequate caregiver education about the illness and knowledge of asthma triggers were barriers to asthma control. Davis et al. (2015) found that direct support workers felt they had not been provided with the education and tools required to manage asthma, including inhaler training. For example, one support worker in this study stated ‘There's no proper training as such to give the inhaler to the client. Somehow that's why I haven't directly helped the client’ (Davis et al. 2015). Although support workers did use asthma management plans to gain information on medication for their clients, they stated these plans were often not fit‐for‐purpose as they lacked clarity and were not concise: ‘we haven't had a clear asthma management plan for this client’ (Davis et al. 2015), therefore acting as another barrier to asthma self‐management.

Only two papers specifically reported barriers to self‐management as perceived by those with intellectual disabilities (Davis et al. 2016a, 2016b). Davis et al. (2016a) reported that participants had concerns regarding side effects of medication, as well as feeling stigma around medication use. Davis et al. (2016b) reported that participants had poor attentiveness and poor comprehension making inhaler training more challenging. Cole (2019) found that patients who attended the specialist asthma clinic had trouble understanding the literature available in typical asthma clinics. It is stated in the report that 90% of the users (people with intellectual disabilities) of the clinic would not manage the asthma plan that is provided by Asthma UK (Cole 2019).

## 4 Discussion

The aim of this scoping review was to consolidate the published evidence relating to self‐management of asthma in those with intellectual disabilities. Common themes have emerged from the results of this review including the lack of research in this area, the importance of family and carers in helping the person with intellectual disabilities self‐manage their asthma, the importance of tailored and easy‐to‐read resources, and the need to overcome specific barriers including lack of asthma education and training for those with intellectual disabilities and their carers.

### 4.1 Lack of Research

One of the key findings of this review relates to the small number of studies found. Six studies were included in the review following the screening process and only five of these were primary research, indicating that this is an under‐researched area. The oldest article was from 2015 which is less than 10 years ago, indicating this is a relatively new area of research. Sheerin et al. (2021) conducted a systematic review of medication management in intellectual disability settings. This review found that while there are some guidelines and proposed practice frameworks, these are not based on a strong evidence base. This reflects the findings of this review in that evidence is limited.

There are existing publications of reviews focused on self‐management of other long‐term conditions such as diabetes in those with intellectual disabilities (Beresford and Kozlowska 2022) and general long‐term condition management (Hanlon et al. 2018). No such reviews exist for asthma and intellectual disabilities indicating a research gap. These reviews highlight similar themes in barriers and enablers of self‐management for these long‐term conditions as found in this review, such as adjustments to account for adequate education and support, more training required for carers, and a person‐centred tailored approach (Beresford and Kozlowska 2022; Hanlon et al. 2018). Beresford and Kozlowska (2022) also highlight that future research should be inclusive and should incorporate patient and public involvement. From the studies found in this review only two directly interviewed and involved those with intellectual disabilities embedded in the study design. The UK Medical Research Council highlights the importance of involving stakeholders in the development of medical intervention development (Skivington et al. 2021). NICE also highlights the importance of patient public involvement in the development of their clinical guidance (NICE 2023). Despite the fact that those with intellectual disabilities have poorer health outcomes than the general population (O'Leary et al. 2018), they have historically been marginalised and excluded from health research (Cardell 2015; Feldman et al. 2014). It is therefore important to include those with intellectual disabilities in future research on self‐management of their asthma.

There was no acknowledgement of potential bias or influence of the relationship between the researcher and participants in any of the studies. It is important to highlight that three of the studies included all had the same primary author, Davis, S., with a lot of overlap in the other listed authors of these studies, indicating the lack of expertise in this field.

### 4.2 Involvement of Carers and Medication Management

The role of carers and support staff may be expected to be different in those with intellectual disabilities who self‐administer their medication to those who rely on others to administer it for them. However none of the papers directly investigated this. What is apparent from the findings of this review is that carers and support staff, along with other health professionals, are extremely important in the empowerment and supporting of those with intellectual disabilities in the self‐management of their asthma. This is not acknowledged in asthma guidelines, and the few papers we reviewed did not clearly investigate their role.

Reflecting the focus of the included papers, this review highlights that much of the support needed to help people with intellectual disabilities manage their asthma is related to medication management. Erickson et al.'s (2016) qualitative study looking at caregivers' views of medication management in those with intellectual disabilities highlighted the complexity around this process. Medicine management involves many steps including obtaining the prescription which requires interaction with a prescribing professional, getting the prescription ‘converted’ into actual medication, and then taking the medication which involves knowing how and when to take it (Erickson et al. 2016). Potentially, many of these steps can inhibit asthma self‐management, consolidating the need for more research on this topic.

Medication self‐management also requires judgment and decision‐making (Crossley and Withers 2009). Huneke et al. (2012) found that medication knowledge in those with intellectual disabilities was better for those patients who had capacity, whereby capacity was defined as having the capacity to consent for medical treatment. This indicates that the level of intellectual disability influences how autonomous they may be in the medication aspect of self‐managing their condition. This review found that caregivers and professionals working with children with an intellectual disability perceived the ability of children to learn to manage their asthma was higher in children with mild‐to‐moderate disability compared to those with severe disability (Nabors et al. 2022). It was also found that support workers have a range of involvement in supporting their clients with asthma and support workers had to decide the degree to which they are able to self‐manage their asthma to provide the correct level of support (Davis et al. 2015). An unrecognised barrier to effective asthma self‐management in this population is challenging carer and family perceptions on when/how the child or adult with intellectual disabilities is able to self‐manage.

### 4.3 Health Literacy and Education

Given how the medication management process is complex (Erickson et al. 2016), ensuring adequate patient education is highly important. This review found that patient education is important and was a recurring overarching theme in the studies found. Lack of patient education was identified as a barrier to self‐management. Empowering people to self‐manage involves educating them about their condition and how to manage it with sufficient medication knowledge and training. Paasche‐Orlow et al. (2005) completed a cohort study of adults hospitalised with asthma and found that inadequate health literacy was associated with lower asthma medication knowledge and poor inhaler technique. Their results indicated that tailored education to teach people about correct interventions can overcome these barriers to self‐management (Paasche‐Orlow et al. 2005). Erickson et al. (2018) found that the most frequently cited barriers to controlling asthma are inadequate education about the illness.

Smith et al. (2019) completed a scoping review looking at whether people with intellectual disabilities understand their medication. This review differs from this current scoping review as it focuses solely on medication and no other aspects of self‐management, and it also features diseases and conditions other than asthma (Smith et al. 2019). This review featured the Davis et al. (2016a) study that was featured in this scoping review. The main results of their findings are that people with intellectual disabilities often lack understanding of their medication and have incorrect knowledge about side effects (Smith et al. 2019). The scoping review also highlighted that accessible information helps to improve knowledge of medication (Smith et al. 2019) which was a result that was found in this scoping review. This review found that certain techniques such as more accessible information in easy‐to‐read formats may enable those with intellectual disabilities to better understand it. It is important to note that Cole (2019) found that in their trial of the dedicated asthma clinic for those with intellectual disabilities, 90% of those who attended the clinic could not manage the asthma plan by Asthma UK. Information should be accessible and tailored to different levels of intellectual disability and cognitive ability. Along with tailored information, the results of this review indicate that visual cues and reminders about taking medication were useful. Davis et al. (2016a) found that mobile phone alarms for medication were helpful. Ramsey et al. (2020) completed a systematic review of digital interventions for paediatric asthma management and found that digital interventions improved medication adherence and health outcomes. Digital interventions could therefore be useful for those with intellectual disabilities, such as a mobile application that can be set up to alert them when to take their medication. Apps have been developed for young people to help them self‐manage their asthma; however, the acceptability and usefulness of such apps are difficult to conclude at present due to a lack of controlled trials and adequate sample sizes (Alquran et al. 2018; Davis et al. 2018).

It is also important to include all of those who are involved in helping and supporting the person with the management of their asthma. From this review it has been found that many carers and support workers feel they lack the knowledge to adequately support those they care for and work with their asthma (Davis et al. 2015; Erickson et al. 2018). Recommendation for clinical practice therefore is to ensure the carers involved have been adequately trained in medication administration and have access to information and resources on asthma.

### 4.4 Strengths and Limitations

One of the main limitations of this scoping review was the time and resource constraints which meant an internet search on unpublished literature was not feasible. Despite this, a quality improvement paper was included (Cole 2019). It is acknowledged that as grey literature was not included in this review, we may have missed some relevant articles or pieces of research. The focus of this paper was on published literature that informed evidence‐based guidelines and is seen as higher quality research in terms of the hierarchy of evidence. It also acknowledged that some search terms for intellectual disabilities were omitted from searches such as ‘mental retardation’ which was used widely in the United States until about 20 years ago; therefore resulting in potential papers published pre‐2015 to be missed.

## 5 Conclusions and Implications

The aim of this scoping review was to look at the evidence that currently exists on the self‐management of asthma in those with intellectual disabilities. Six relevant studies were included. Lack of education, the importance of family and carers, medication management, and the importance of adequate training were common themes across these studies. The key take‐home from the review is the lack of research that currently exists in this area, and the relative infancy of the research that has been conducted. This indicates that there is a research gap and highlights the need for future research on how to better empower and support those with intellectual disabilities in self‐managing their asthma. The next study will address these gaps. Given the lack of current evidence, it will be important to gain the views and experiences of those with intellectual disabilities and asthma to ensure findings are relevant for this population. The study also highlights that families and carers must be involved in this research as they play an important role in supporting those with intellectual disabilities.

## Funding

This scoping review has been completed as part of a PhD project funded by Edinburgh Napier University.

## Conflicts of Interest

The authors declare no conflicts of interest.

## Data Availability

Data sharing not applicable to this article as no datasets were generated or analysed during the current study.
